# Cortical injury in multiple sclerosis; the role of the immune system

**DOI:** 10.1186/1471-2377-11-152

**Published:** 2011-12-06

**Authors:** Caroline A Walker, Anita J Huttner, Kevin C O'Connor

**Affiliations:** 1 Department of Neurology, Yale School of Medicine, 15 York Street, PO Box 208018 New Haven, CT 06520-8018, USA; 2 Department of Pathology, Yale School of Medicine, 310 Cedar Street LH 108 PO Box 208023, New Haven, CT 06520-8023, USA; 3 Human and Translational Immunology Program, Yale School of Medicine, The Anlyan Center for Medical Research & Education, 300 Cedar Street P.O. Box 208011, New Haven, CT 06520, USA

## Abstract

The easily identifiable, ubiquitous demyelination and neuronal damage that occurs within the cerebral white matter of patients with multiple sclerosis (MS) has been the subject of extensive study. Accordingly, MS has historically been described as a disease of the white matter. Recently, the cerebral cortex (gray matter) of patients with MS has been recognized as an additional and major site of disease pathogenesis. This acknowledgement of cortical tissue damage is due, in part, to more powerful MRI that allows detection of such injury and to focused neuropathology-based investigations. Cortical tissue damage has been associated with inflammation that is less pronounced to that which is associated with damage in the white matter. There is, however, emerging evidence that suggests cortical damage can be closely associated with robust inflammation not only in the parenchyma, but also in the neighboring meninges. This manuscript will highlight the current knowledge of inflammation associated with cortical tissue injury. Historical literature along with contemporary work that focuses on both the absence and presence of inflammation in the cerebral cortex and in the cerebral meninges will be reviewed.

## Review

### Introduction

Multiple sclerosis (MS) is widely viewed as a disease of white matter [1]. White matter lesions that include demyelination and neuronal damage are readily visible by MRI and macroscopically upon autopsy [2,3]. White matter lesions visualized via MRI are used to diagnose MS, in effect making these lesions the leading pathognomonic sign for MS [4]. The most widely accepted animal model, experimental autoimmune encephalomyelitis (EAE) in rodents, is based on an induced autoimmune reaction against myelin proteins of white matter of the central nervous system (CNS) [5]. That such injury is easily identifiable and ubiquitous, white matter pathology has been the subject of considerable attention. Although white matter damage is clearly present in the disease, it is not the only site within the CNS where the pathology of MS occurs. The cerebral cortex of the MS brain has recently been recognized as a major site of disease pathogenesis, perhaps now moving toward equal importance as the white matter. This is not to say that tissue damage in the cortex was never recognized. Gray matter damage has been described in MS since the earliest known reference to the disease phenotype. In *Pathological Anatomy *(1838), the Scottish pathologist Robert Carswell describes and illustrates a spinal cord that is viewed, by medical historians, to be among the very first documented cases of MS [6,7]. In this report Carswell notes the presence of lesions and atrophy. Regarding the grey matter damage, he writes, "The depth to which the medullary substance was affected in this matter varied from half a line to three or four lines, and on dividing the cord, it was seen to penetrate as far as the gray substance." His illustrations of the spinal cord's traverse sections demonstrate lesions exclusive to the white matter and those that have extended from the white into the gray matter. Although MS was not named a separate disease until 30 years later in Jean-Martin Charcot's *Histology de la Sclerose en Plaque *(1868), in 1838 Carswell recognized that the currently unclassified CNS pathology, which he described, was not restricted to the white matter. In this review, we summarize the present-day knowledge of the role that the immune system plays in MS cortical tissue damage, focusing on the cellular and molecular characteristics of the immune infiltrate found within the cortex and meninges.

### Characteristics of cortical lesions

Despite acknowledgement in the early studies of MS and that the disease includes cognitive symptoms, cortical involvement in MS has been given less attention than the characteristic white matter lesions until recently. Given that cortical damage is now recognized as a major site of disease pathology, why has this occurred? The most plausible explanation is that cortical lesions are simply not obvious by the standard means of visualization (MRI and histopathology) and early macroscopic studies suggested that they represent a minor fraction of damage that occurs in the brain [8]. Cortical immune infiltrates associated with tissue damage are often sparse [9]. In the absence of an immune infiltrate, these lesions maintain a normal water concentration and therefore are not hyperintense on T2 weighted MRI like white matter lesions [10] highlighting why they are not easily visualized. Although identification of individual cortical lesions in MS are elusive, cortical atrophy in patients with MS is apparent, particularly in the hippocampus [11]. It has been established that the cortex atrophies more rapidly than white matter in patients with MS and that the degree of cortical atrophy is independent of that which occurs in the white matter [12]. Cortical atrophy correlates with the clinical progression of the disease better than the white matter lesion load [13-15]. These findings support the idea that cortical damage may better explain the symptoms of cognitive impairment associated with MS, such as anterograde memory loss, whereas white matter lesions manifest clinically as motor deficits [11]. There is not an appreciable correlation [16,17] between white matter lesion load and cortical tissue damage, as measured by imaging and histochemistry, indicating that the pathological processes may, to some extent, occur independently. Cortical lesions have been classified using a number of systems [18-20]. All of the systems share similar descriptions of the three major lesion types [21]. These are identified as type I (contiguous with subcortical white matter lesions), type II (exclusively intracortical and extending through all the cortical layers), and type III (extended from the pial surface to the superficial cortical layers). White matter lesions in chronic MS are often characterized by severe BBB breakdown and often include a lymphocytic infiltrate. Interestingly, cortical lesions from the same subject lack detectable plasma/serum-derived proteins and basement membrane alterations indicating that the BBB disruption is not always associated with intracortical demyelination in progressive MS [22].

The examination of inflammation in MS brain tissue principally includes marking for T cells, B cells, dendritic cells, microglia and macrophages. T cell subsets such as T helpers (CD4+), cytotoxic (CD8+), and memory T cells, which play different roles, are also of interest. B cells are examined less often and plasma cells are often not included in such analyses. It is useful, however, to include markers for plasma cells that may secrete pathogenic autoantibody. Identifying B cells is important as they may function as very effective antigen presenting cells (APC) in MS and their ablation results in decreased lesion load [23]. Application of such immunohistochemistry to cortical tissue has highlighted a further contrast with most white matter lesions. That is, cortical lesions in progressive MS harbor considerably less inflammation than that which is observed in the white matter. It is important to point out that the absence of ongoing active cortical demyelination is often accompanied by a paucity of infiltrating immune cells. Active cortical demyelination can be identified by the presence of recent myelin degradation products in macrophages/microglia. When such active lesions are examined, more inflammation is observed, but this inflammation is much less pronounced than that in the white matter. Sparse infiltrates associated with inactive demyelination does not usually differ with control areas in MS brain that are not damaged [17,20,24]. It should be pointed out that regions of MS brain that are free of injury often harbor immune cells. Activated effector memory T cells (T_EM_), B cells and T cells reside in the white matter and cortical tissue that appears to be free of lesions [25-27]. The cortical lesions that include few detectable infiltrates, sharply contrast those of the white matter lesions from the same brain that have conspicuous infiltrates [28] indicating, perhaps, that the location of a lesion may influence the immune response. The little inflammation that is present is variable depending on the type of cortical lesion. Lesions that extend through the white matter and cortex (type I) and deep cortical lesions have higher counts of inflammatory cells than those that are exclusively intracortical [29,30], but both have significantly less inflammation than those of white matter from the same brain [31]. The minor infiltrate in the cortical lesions include activated microglia, scarce myelin-laden macrophages, CD3+ T cells, CD20+ B cells, and rare CD138+ plasma cells. Interestingly, microarray-based gene expression profiling has revealed that immunoglobulin-related genes are upregulated in the cortex of progressive MS specimens [32]. These data appear to stand in contrast to the immunohistochemistry-based studies that suggest inflammation is not a characteristic of cortical tissue damage. Further examination of the same specimens used in the microarray work showed that both plasma cells, which robustly express immunoglobulin, and some B cells present in the meninges were likely contributing to the variation in expression.

### Cortical injury associated with inflammation

To date, a modest amount of data has been presented to support a role for inflammation in cortical tissue damage. However, a picture contesting the view that cortical demyelination occurs in the absence of inflammation is beginning to emerge. Indeed, a recent study using biopsy material derived from early MS (diagnosis confirmed through follow up) clearly demonstrates that demyelination of pure cortical tissue includes plainly evident inflammation [33]. Here, infiltrating macrophages associated with cortical tissue damage contained products of degraded myelin such as PLP and CNPase. Lymphocytes were present in both the exclusively parenchymal and the perivascular regions. This infiltrate included T cells, B cells and antibody-producing plasma cells. The CD4+ T cell population (determined by estimating the number of CD3+ cells not stained with CD8), were less abundant than CD8+ T cells. In addition to demyelination, neuronal and axonal damage was also recorded. Interestingly, evidence for white matter demyelination via MRI was not observed at this early stage of the disease, suggesting that cortical tissue damage may precede tissue injury in the white matter. That more progressive and/or chronic forms of MS can include cortical damage that may not be associated with inflammation, raises questions regarding the possibility that resolution of cortical inflammation in some stages of MS may occur. A growing number of models of MS include intracortical lesions with extensive demyelination associated with inflammation [34-36]. In one such recently described rodent model [37], the cortical inflammatory infiltrate was shown to considerably diminish shortly after establishment of the tissue damage suggesting that a similar course of resolution may occur in some stages of the human disease.

So, it seems that early MS may include cortical damage associated with considerable inflammation while progressive stages harbor less cortical inflammation. Why have these vast differences in MS cortical inflammation only come to light recently? It is important to bear in mind that much of the research that requires MS CNS tissue shares a common denominator, that is many studies are largely focused on progressive disease. Such bias is often unavoidable in MS research because the majority of clinical specimens available for research are collected nearly invariably from autopsy with more rare collections derived from biopsy. This, of course, results in an unavoidable, but evident sampling bias. Furthermore, the majority of autopsy specimens are derived from progressive late-stage disease and biopsies are often derived from the early stages of disease, which present a very different pathological course. Thus, much of the disease spectrum is not well represented. Larger studies, with specimens derived from multiple centers are needed to more precisely understand the relationship between cortical and white matter inflammation at different stages of the disease.

### Cortical demyelination and inflammation: meningeal lymphocytes

Follicles of the lymph nodes contain germinal centers populated by antigen-activated B cells. Within this structure, B cells undergo antigen-driven clonal expansion, affinity maturation and differentiation into memory B cells and plasma cells. Among the support structure required for this to occur are T cells and follicular dendritic cells (FDC). FDCs present antigen and provide survival and proliferation signals to B cells. These cells also produce a B cell chemoattractant, CXCL13, which regulates migration of B cells. FDC can be recognized by their expression of CD35. In several autoimmune diseases, such as rheumatoid arthritis (RA) [38], substantial lymphocyte infiltrations are seen at the site of autoimmune-mediated tissue damage. These infiltrates, which are not associated with lymphoid tissue, often form germinal center (GC) -like structures [39] where the presence of FDCs as well as T and B cells have been demonstrated. In addition to RA, such ectopic GCs or ectopic lymphoid aggregates are observed in the tissue of patients with RA, Sjogren's syndrome, Crohn's disease and Hashimoto's thyroditis [40-42] and within some tumors [43-46]. These structures are thought to be a source of the autoreactive B cells and antibodies and participate in the maintenance of the autoimmune response, although more investigation is needed to confirm this.

Meningeal inflammation in the MS CNS, which has been the subject of much investigation in the last decade, was described in the early twentieth century [47]. Additional early studies of MS tissue report that inflammatory changes affected not only the leptomeninges and arachnoid, but also dura mater. The inflammation described in these reports was either acute or chronic and often led to thickening and fusion of the leptomeninges with the dura [48,49]. The presence of meningeal inflammation in some animal models of MS has also been described [50,51]. Among the more contemporary reports that describe inflammation in the MS CNS that appeared to adopt an organized morphology, is that of Prineas [52]. Here, white matter-associated perivascular spaces holding lymphocyte containing capillaries and plasma cells resemble lymph node architecture. In addition, Guseo and Jellinger in 1975 described the infiltrates that they found to populate the meninges and deep sulci of MS brain as "grouped" [53]. These organized structures present in the meninges of the MS CNS have been more clearly defined in the last decade [54]. They resemble the B cell follicles observed in autoimmune tissue and neoplasms and appear to be exclusive to the meninges, as they are not found at the site of parenchymal lesions. Meningeal infiltrates in MS differ from those found in perivascular regions of the parenchyma; the network of follicular dendritic cells is not present in the parenchyma, clusters of proliferating B cells appear exclusively in the meninges, as does the expression of lymphocyte homing chemokines. They are not exclusive to MS [55], but do seem rare in cases of inflammatory CNS disease. Larger studies are needed to more clearly define their distribution.

B-cells, T-cells, plasma cells, and a supporting network of follicular dendritic cells that imitate those in secondary lymph nodes populate these follicles. These lymphocytes are often associated with meningeal blood vessels. Meningeal follicles emulate peripheral lymphoid germinal centers in that proliferating B cells, evidenced by Ki67 expression, collect within the structure and lymphocyte homing chemokines such as the chemoattractant CXCL13 expressed by CD35 positive FDCs have been observed in a subset of patients [54]. These follicles, however, are incomplete when compared to those found in secondary lymphoid organs. They do not own all of the characteristic structures, such as HEVs, or all of the homing chemokines, such as CCL21 or PNad. Meningeal B cell follicles were evident in about half of secondary progressive MS cases examined in one study [56] but rare or absent in primary progressive cases reported in another study by the same team [55]. Another study reported that these meningeal structures are present in both primary and secondary progressive disease [9] and that an active disease process correlated with their presence. Many progressive cases can include a modest meningeal immune cell infiltrate that do not include B cell follicle structures (lack organization) [55]. Thus in general terms, there appears to be a gradient of meningeal inflammation that ranges from absent to moderate and diffuse and then to that which includes the formation of follicle-like aggregates. This gradient is also reflected in the severity of active demyelination and tissue damage. Of course, whether tissue without or with moderate inflammation had previously harbored follicles that have resolved is not known. Larger, and certainly more complex studies will be required to determine if such follicles appear in relapsing remitting MS before the transition to late-stage or progressive disease has occurred. Imaging techniques are likely to be required for longitudinal studies that are not possible with autopsy-derived specimens.

Analysis of whole bi-hemispheric sections clearly illustrates that the B cell follicle-like structures are numerous and found widely distributed throughout meninges and most often reside in the deep infoldings of the cerebral sulci [57]. They do however vary considerably in number of cells per structure and in structures per case. Specimens harboring follicles often include B cell and plasma cell infiltrates in white matter lesions, whereas those without follicles typically have fewer white matter associated B cell infiltrates. There appear to be regions of meningeal inflammation, which are not associated with tissue damage [58], but there are many instances where the two are closely linked. Increased cortical lesion load, in terms of quantity and extent of demyelination, correlates with the presence of meningeal follicles, as does the preponderance of subpial (type III) lesions (extended from the pial surface to the superficial cortical layers). Follicles are most often found adjacent to these subpial lesions. These collective data, of course, suggest that the formation of follicles is related to or plays a role in damage of the cortical tissue. The presence of follicles also correlates with increases in cortical atrophy and loss of neurons, astrocytes, and oligodendrocytes [55]. The tissue damage occurs in a gradient that begins at the pial surface then decreases with distance from this region. Cytotoxic factors diffusing from the meningeal compartment are suspected to play a role in this damage. The degree of disease course severity also appears to correlate with the presence of organized meningeal inflammation, whether such formation is contributory or a consequence of the severity remains to be determined.

It is clear from these data that a compartmentalized B cell response occurs within the MS CNS. Many stages of B cell differentiation that are usually observed only in secondary lymphoid organs appear to occur, suggesting a favorable microenvironment is arranged in the CNS. This proliferation of B cells in the MS CNS has been attributed to EBV infection [59,60], but this has been considerably controversial because a number of groups have found that EBV+ B cells are not overrepresented in the MS CNS [32,61-63]. Thus, the question remains as to whether peripherally activated B cells are selectively recruited to the CNS tissue or if B cells are recruited and then mature locally and differentiate into plasma cells emulating a germinal center. Interestingly, a portion of B cells that populate white matter parenchymal lesions are clonally related to those in meninges, and both of these populations have clonal siblings represented in the CSF [27]. And the CSF IgG that comprises the characteristic oligoclonal bands are derived from this network of CNS resident B cells [64,65]. It remains to be understood how this network is established in terms of its origin. That cervical lymph nodes harbor brain-derived antigens [66] suggests that cells that comprise this network can be associated with the periphery. However, such antigens appear only after tissue damage has occurred hence it remains possible that these cells first experience antigen in the CNS.

### Cortical inflammation in models of MS

A thorough review of models that emulate MS cortical pathology is beyond the scope of this review, however there are studies that highlight the findings in the human disease and guide questions for future study. For example, it is interesting to note that MS models emulating cortical tissue damage not only harbor immune infiltrates of T cells, B cells and macrophages, but also appear to depend on antibodies and complement. A rat model of MS displayed extensive cortical demyelination associated with deposition of immunoglobulin on myelin sheaths [35]. Similarly, a non-human-primate model for MS [34] revealed immunoglobulin and complement c9 deposition in regions of cortical demyelination. Derfuss et al. developed a mouse model for gray matter damage in MS in which immune damage mediated by TAG-1 (contactin-2 homologue)-specific T cells resulted in gray matter inflammation in the spinal cord and cortex. When the TAG-1 specific T cells were co-transferred with a monoclonal antibody against myelin oligodendrocyte glycoprotein (MOG), focal perivascular demyelination occurred in the cortex. They also found contactin-2 specific T cells and antibodies in MS patients, suggesting that an autoimmune response against the protein may be related to MS gray matter pathology [67]. Similarly, Huizinga et al. produced an MS mouse model that exhibits axonal loss and cortical lesions by inducing autoimmunity against neurofilament light (NF-1). These models, along with the rat and primate models, support the idea that direct immunological damage to the cortical tissue plays a role in the observed pathology [68]. There is also evidence that more indirect immune-mediated damage to gray matter contribute to the pathology of MS. Centonze and colleagues suggest that cortical damage could be caused, in part, by collateral damage of pro-inflammatory cytokines released by immune mediators reacting to myelin proteins. Here, cytokines, such as IL-1β, TNFα, and INFγ, released by T cells and microglia increase AMPA receptor activity on neurons, contributing to neuronal damage and cortical pathology in models of MS [69].

## Conclusions

Future work is necessary to more clearly define the emerging picture of immune-associated cortical demyelination that occurs in MS. Understanding the relationship between the profound inflammation commonly seen in the white matter and that of the cortex, which seems to be less consistently observed, is certainly a priority. Many other questions remain: From where do the meningeal infiltrates arise? Do the cells that populate these structures emerge from the periphery then migrate to this compartment or do pioneer naïve cells experience antigen in the CNS then proliferate exclusively within this compartment? How exactly do they affect tissue damage? Are these cells autoreactive? The antigen(s), whether they are self, environmental or unique to individuals, unquestionably need to be defined. While important recent findings have strengthened our understanding of MS cortical tissue damage they also highlight the critical need to further understand cortical pathology and pathogenesis.

## List of abbreviations used

(AMPA): 2-amino-3-(5-methyl-3-oxo-1,2- oxazol-4-yl)propanoic acid; (CNPase): 2',3'-Cyclic-nucleotide 3'-phosphodiesterase; (APC): Antigen presenting cell; (BBB): Blood brain barrier; (CNS): Central nervous system; (CCL21): Chemokine (C-C motif) ligand 2; (CXCL13): C-X-C motif chemokine 13; (EBV): Epstein-Barr virus; (FDC): Follicular dendritic cell; (GC): Germinal center; (HEV): High endothelial venules; (INFγ): Interferon *γ*; (IL-1β): Interleukin-1β; (MRI): Magnetic resonance imaging; (MS): Multiple Sclerosis; (MOG): Myelin oligodendrocyte glycoprotein; (NF-1): neurofibromin-1; (PNad): peripheral node addressin; (PLP): Pyridoxal-phosphate; (RA): Rheumatoid arthritis; (TAG-1): Transiently expressed axonal glycoprotein 1; (TNFα): Tumor necrosis factor α.

## Competing interests

The authors declare that they have no competing interests.

## Authors' contributions

CAW, AJH, and KCO researched the literature and wrote the manuscript. All authors have read and approved the final manuscript.

## Pre-publication history

The pre-publication history for this paper can be accessed here:

http://www.biomedcentral.com/1471-2377/11/152/prepub
